# Lung cancer screening eligibility and completion among adults diagnosed with lung cancer: retrospective analysis of 2024 National Health Interview Survey

**DOI:** 10.1007/s10552-026-02229-x

**Published:** 2026-08-19

**Authors:** Izabella Zanazanian, Angela Yin Chieh, Aarushi Madan, Franceskrista Morales, Gelareh Sadigh

**Affiliations:** Department of Radiological Sciences, University of California Irvine, 101 The City Dr S, Orange, Orange, CA 92868 USA

**Keywords:** Lung cancer screening, Low-dose CT, USPSTF, Screening eligibility, Health disparities

## Abstract

**Purpose:**

Lung cancer screening using low-dose computed tomography (LDCT) is recommended for high-risk individuals, yet uptake remains low. We evaluated screening eligibility and completion among adults with lung cancer.

**Methods:**

In the 2024 National Health Interview Survey, adults aged ≥ 50 years with a lung cancer diagnosis within the past 10 years were included. Eligibility at diagnosis was determined using 2021 United States Preventive Services Task Force criteria based on self-reported smoking history. LDCT completion before diagnosis was assessed. Weighted multivariable logistic regression identified factors associated with outcomes.

**Results:**

115 participants were included (weighted *n* = 638,702). Eligibility could not be determined for 3.5% (4/115). Among those with known eligibility, 52.3% (58/11) met screening criteria at diagnosis. Female sex (OR 0.31; 95% CI 0.12–0.77; *p* = 0.01) was associated with lower odds of eligibility, while non-white race showed a trend toward lower eligibility (OR 0.35; 95% CI 0.12–1.04; *p* = 0.06). After excluding participants who never smoked, no variables were significant. Overall, 83.6% (92/110) reported receipt of LDCT, but only 32.7% (36/110) received it before diagnosis. No variables were significantly associated with screening completion.

**Conclusion:**

Among US adults with lung cancer, approximately half were screening-eligible, and only one-third underwent screening before diagnosis, highlighting gaps in both eligibility and utilization.

**Supplementary Information:**

The online version contains supplementary material available at https://doi.org/10.1007/s10552-026-02229-x.

## Introduction

Lung cancer is the third most diagnosed cancer and leading cause of cancer-related death in the United States [1, 2]. Early detection through low-dose computed tomography (LDCT) is shown to decrease mortality by 20% in high-risk individuals [3]. The most recent US Preventive Services Task Force (USPSTF) lung cancer screening (LCS) guidelines in 2021 expanded eligibility for LDCT [4] to include adults 50–80 years of age (previously 55–80) [5] with 20 pack-year smoking history (previously 30 pack-year) [5] who currently smoke or quit smoking within the last 15 years [4]. While the rate of LCS has somewhat increased over the last decade, uptake remains low (< 22%) and is exacerbated by socioeconomic disparities [6, 7].

Previous studies have shown that women, Black, and Hispanic patients diagnosed with lung cancer were less likely to be LCS-eligible, resulting in a health disparity [8, 9]. This might be due to LCS eligibility criteria being strictly limited to age and smoking history, and the need for a more accurate and inclusive risk prediction model [10]. However, most of this evidence is derived from multicenter cohort studies that are geographically limited and may not fully capture national patterns. In contrast, nationally representative analyses have largely focused on populations without lung cancer, leaving a critical gap in understanding how eligibility criteria perform among those ultimately diagnosed with the disease [11, 12].

Aside from disparities in LCS eligibility, a variety of patient-level (e.g., stigma, fear, lack of awareness), provider-level (e.g., limited time, unfamiliarity with guidelines), and system-level (e.g., incomplete smoking data in electronic health records) barriers also contribute to the low utilization of LCS [10, 13]. These barriers disproportionately affect populations that are already underserved, contributing to persistently lower screening utilization among racial and ethnic minorities and individuals with lower socioeconomic status [14].

Together these gaps highlight the need for studies that use nationally representative data to evaluate disparities in screening eligibility among patients with lung cancer and better understand differences in screening uptake. Using the 2024 National Health Interview Survey (NHIS), we examined rates of LCS eligibility and LDCT completion in patients who were recently diagnosed with lung cancer. We further evaluated demographic and socioeconomic factors associated with LCS eligibility and LDCT completion.

## Methods

This publicly available survey study did not use any private identifiable information and thus did not constitute human subject research requiring Institutional Review Board oversight. An administrative exempt self-determination was filed with our institutional IRB.

### Study design & data collection

A cross-sectional analysis of data from the 2024 NHIS was conducted. The 2024 NHIS collected data from January 1 through 31 December 2024, using a stratified cluster sample designed to represent civilian noninstitutionalized adults aged 18 years and older residing in the 50 States and the District of Columbia. NHIS survey responses were primarily collected using computer-assisted personal interviewing during face-to-face interviews, with an additional option for telephone interviews. The final Sample Adult response rate was 47.9%, calculated using the American Association for Public Opinion Research Response Rate 2 definition [15].

### Study population

The study population included adults aged 50 and older with a lung cancer diagnosis within the last ten years at the time of interview. The age restriction was applied to align the analytic cohort with the age specified in the 2021 USPSTF LCS recommendations.

### Outcomes

The primary outcome was LCS eligibility at the time of lung cancer diagnosis. Patients were classified as LCS-eligible if they reported a history of smoking and either currently smoked or had quit within 15 years before diagnosis if they were a former smoker and had a calculated smoking pack-years of 20 or greater.

The secondary outcome was LDCT completion prior to lung cancer diagnosis, which was classified based on response to, “Have *you ever had a CT scan for lung cancer screening?*” and the date of the reported LDCT in relation to lung cancer diagnosis.

Demographic data such as age, sex, race, ethnicity, language spoken at home, marital status, education, health insurance type, and employment status were collected. The 2013 NCHS Urban–Rural Classification Scheme for counties was used to determine whether individuals lived in nonmetropolitan or metropolitan regions of various sizes [16]. Household income was recorded as a ratio of family income to the poverty threshold and categorized as one of the following: 0–1.29 (low income), 1.30–2.99 (middle income) and > 2.99 (high income) [17, 18]. All sociodemographic data were collected at the time of survey response, and for variables that are subject to change (e.g., employment, income, insurance, marital status, and residence) such information was not available for the time of lung cancer diagnosis.

### Statistical analysis

To generate nationally representative estimates, we used the NHIS Sample Adult final annual weight (WTFA_A), which accounts for unequal probabilities of selection and survey nonresponse, as previously described [19] Descriptive statistics were used to summarize the sample’s sociodemographic and clinical characteristics, as well as study outcomes.

Weighted proportions were calculated to determine rates of LCS eligibility and LDCT completion. Weighted univariate logistic regression analyses were performed to assess the association between nonmodifiable demographic variables and both patients’ LCS eligibility and LDCT completion. Associations between modifiable variables and the outcomes were not evaluated because these characteristics were measured at survey completion, whereas lung cancer diagnosis could have occurred up to 10 years earlier, and therefore may not accurately represent patients’ characteristics during the relevant screening period. Although ethnicity and language were available in NHIS, only two Hispanic and 5 non-English-speaking participants met the study inclusion criteria. Because analyses based on such sparse data yielded unstable and unreliable estimates, ethnicity and language were not included in the regression analyses.

A weighted multivariable logistic regression model was used to assess the association between demographic variables and LCS eligibility. Ethnicity and language were excluded from the model due to small cell sizes in the unweighted sample, which could compromise the model stability. Both variables were also not statistically significant in univariable analysis (*p* > 0.05). No statistically significant interaction between variables was identified. A sensitivity analysis of factors associated with LCS eligibility was performed excluding participants who never smoked. All data analysis was conducted using the STATA software package (Stata/MP 18.0 for Windows, StataCorp, TX). Statistical significance was defined as a *p*-value of less than 0.05.

## Results

This study included 115 participants, representing a weighted population estimate of 638,702 US adults. The mean age was 72.6 years in the unweighted sample, ranging from the ages of 50 to 84 years. Racial background included 1.8% (2/114) Asian, 15.8% (18/114) Black, 80.7% (92/114) White, and 1.8% (2/114) other races. A total of 1.7% (2/115) were of Hispanic or Latino ethnicity. Baseline characteristics of the study population are summarized in Table 1.

**Table 1:** Characteristics of study population

Characteristics	Non-weighted statistics (*n* = 115)	Weighted statistics (*n*** = **638,702)
Age, mean (SD)	72.6 (8.2)	72.3 (8.5)
Sex *n* (%)	Missing = 0	Missing = 0
Female	56 (48.7)	312,906 (49)
Male	59 (51.3)	325,796 (51)
Race *n* (%)	Missing = 1	Missing = 2,366
Asian	2 (1.8)	15,757 (2.5)
Black	18 (15.8)	106,290 (16.7)
Other^1^	2 (1.8)	5,637 (0.9)
White	92 (80.7)	508,651 (80)
Ethnicity, *n* (%)	Missing = 0	Missing = 0
Hispanic or Latino	2 (1.7)	11,072 (1.7)
Not Hispanic or Latino	113 (98.3)	627,630 (98.3)
Language spoken at home	Missing = 7	Missing = 45,896
English	103 (95.4)	556,290 (93.8)
Other	5 (4.6)	36,518 (6.2)
Marital status, *n* (%)	Missing = 8	Missing = 53,339
Married or living with partner	47 (43.9)	373,378 (63.8)
Divorced, separated, single	60 (56.1)	211,985 (36.2)
Employment status, *n* (%)	Missing = 7	Missing = 45,895
Worked in the last week	11 (10.2)	67,698 (11.4)
Did not work in the last week	97 (89.81)	525,109 (88.6)
Income: poverty ratio, *n* (%)	Missing = 0	Missing = 0
Low income	28 (24.4)	118,326 (18.5)
Middle income	45 (39.1)	249,086 (39.0)
High income	42 (36.5)	271,290 (42.5)
Education, *n* (%)	Missing = 0	Missing = 0
At least some college	56 (48.7)	319,160 (50.0)
High school/general education degree or less than high school	59 (51.3)	319,542 (50.0)
Insurance, *n* (%)	Missing = 1	Missing = 1,912
Commercial	34 (29.8)	188,355 (29.6)
Public insurance	53(46.5)	278,541 (43.7)
Other insurance	15 (13.2)	85,734 (13.5)
Uninsured	12 (10.5)	84,161 (13.2)
Urban–rural classification, *n* (%)	Missing = 0	Missing = 0
Non-metropolitan	41 (35.7)	134,656 (21.1)
Metropolitan	74 (64.4)	504,046 (78.9)
Smoking status	Missing = 4	Missing = 29,866
Current	38 (34.2)	200,148 (32.9)
Former	52 (46.9)	277,710 (45.6)
Never	21 (18.9)	130,978 (21.5)

^1^Other includes American Indian/Alaska Native, multiple races, and other races

### LCS eligibility at time of lung cancer diagnosis

LCS eligibility could not be determined for 3.5% (4/115) of the population (weighted proportion: 4.6%) due to missing smoking data needed to determine eligibility. Among individuals for whom LCS eligibility could be determined, only 52.3% (58/111) were eligible at the time of lung cancer diagnosis (weighted proportion: 52.0%). After excluding participants who never smoked, 64.4% (58/90) were LCS-eligible (weighted proportion: 66.3%). Supplementary Table 1 presents the univariable and multivariable logistic regression analyses examining nonmodifiable demographic factors associated with LCS eligibility at the time of lung cancer diagnosis using survey weights. The adjusted model for LCS eligibility showed that female sex (vs. male) (OR 0.31; 95% CI 0.12–0.77; *p* = 0.01) was associated with lower odds of being eligible for LCS. Further, non-White patients were marginally less likely to be eligible for LCS (OR 0.35; 95% CI 0.12–1.04; *p* = 0.06).

In a sensitivity analysis excluding people who never smoke, none of the variables were significantly associated with LCS eligibility (Supplementary Table 1). Comparing participants’ demographics based on their smoking status, we observed 76.2% (16/21) of participants with lung cancer who never smoked were female compared to 43.3% (39/90) of participants with a history of smoking smoke (*p* = 0.007). Further, female participants had lower mean pack-years compared to male participants (37.9 vs. 56.1; *p* = 0.01). Females also on average had longer time since smoking cessation than men (20.2 vs. 11.3; *p* = 0.01). We did not observe any statistical racial difference based on smoking status, pack-year or quite year.

### LDCT completion

Among patients with lung cancer, regardless of LCS eligibility at time of diagnosis, 83.6% (92/110) reported ever receiving an LDCT (weighted proportion: 83.6%); however, only 32.7% (36/110) reported that the LDCT occurred prior to their diagnosis (weighted proportion: 29.6%). In univariable logistic regression analyses no demographic factor was associated with LDCT use prior to lung cancer diagnosis (Supplementary Table 2).

## Discussion

In this nationally representative study of U.S. adults aged 50 years or older diagnosed with lung cancer, we found that approximately half of patients (52%) and two-thirds (64%) of participants with a history of smoking met USPSTF 2021 LCS eligibility criteria at the time of diagnosis. In multivariable analysis, female sex was associated with significantly lower odds of meeting screening eligibility criteria, and non-white race had a non-significant but marginal association with lower odds of LCS eligibility. These findings suggest that current screening guidelines may not capture all individuals who develop lung cancer. Additionally, although most patients reported undergoing LDCT at some point, fewer than one-third received LDCT prior to diagnosis. Together, these findings highlight gaps in both screening eligibility and completion.

Eligibility for LCS under USPSTF guidelines is determined primarily by age and smoking history [4]. While expansion of the guidelines in 2021 aimed to broaden access, our results suggest that a substantial proportion of individuals who develop lung cancer remain ineligible at the time of diagnosis. A prior study has similarly reported only 43% of patients with lung cancer who would have met screening eligibility under the 2021 USPSTF criteria [11].

Additionally, prior literature has shown disparities in LCS eligibility, with women and racial and ethnic minority populations being less likely to meet 2021 USPSTF LCS criteria both in general population [8, 9] and among those diagnosed with lung cancer [11, 12]. However, these studies largely focused on individuals with a history of smoking. In our study, women had significantly lower smoking pack-years and longer time since smoking cessation than men, which may partially contribute to the observed sex disparities in LCS eligibility. We also found that disparities by sex were no longer observed after excluding participants who never smoked. This finding is explained by higher proportion of women among patients with lung cancer who never smoked and is consistent with prior studies [20]. Our results also showed a marginal disparity in LCS eligibility based on race, which also did not persist after exclusion of participants who never smoked. Although we did not observe significant racial differences in smoking status, pack-year, or quit year, our analyses were limited by the small sample size, which required dichotomization of race as White versus non-White. Previous studies have also reported a higher incidence of lung cancer among Asian individuals who never smoked, which may similarly contribute to disparities in LCS eligibility [21, 22]. Overall, these findings suggest that reliance on age and pack-year thresholds may fail to identify at-risk individuals, particularly those with lower cumulative smoking exposure or who never smoked.

A central finding of this study is the low proportion of patients who reported receiving LDCT prior to lung cancer diagnosis. Although 83.6% of patients reported having undergone CT imaging at some point, this likely reflects imaging performed for a variety of clinical indications rather than formal lung cancer screening. In contrast, only 32.7% reported LDCT prior to diagnosis, suggesting that relatively few patients underwent screening. A recent analysis of the 2024 NHIS reported a national LCS rate of 18.3% among adults meeting the 2021 USPSTF eligibility criteria [23]. Although the uptake observed in our study was slightly higher (32.7%), our cohort consisted of individuals who were subsequently diagnosed with lung cancer and therefore may have had higher utilization of LDCT compared with general population. Nevertheless, our findings continue to highlight a substantial gap between screening eligibility and screening completion and are consistent with prior reports demonstrating persistently low uptake of LCS in the United States [6, 24].

Several factors may contribute to these gaps. Patients may conflate screening LDCT with diagnostic or surveillance CT imaging obtained during evaluation or management of lung cancer, as prior studies have shown limited patient understanding of distinctions between screening and diagnostic imaging modalities [25]. Lung cancers may also be incidentally detected on CT scans performed for nonscreening indications, a phenomenon that has been well described in prior observational studies [26]. Additionally, underreporting smoking history in survey-based data may result in misclassification of screening eligibility [27]. Together, these factors may contribute to discrepancies between self-reported LDCT and the true prevalence of screening LDCT.

Although personalized or risk-based screening models [28] have been proposed as alternatives to fixed age- and smoking-based criteria, these approaches often require numerous clinical, behavioral, and sociodemographic variables that may not be consistently available or accurately captured in routine clinical practice or population-based datasets. As the number of required inputs increases, the likelihood of missing or incomplete data also rises, potentially limiting real-world feasibility and scalability.

### Limitations

Several limitations should be considered when interpreting these results. First, the use of self-reported survey data introduces the potential for recall bias and misclassification, particularly regarding smoking history and timing of LDCT. Second, patients may not distinguish screening LDCT from diagnostic CT imaging with sufficient granularity, which may lead to overestimation of screening completion. Third, the cross-sectional design precludes causal inference. Fourth, we were only able to evaluate associations between the outcomes and nonmodifiable demographic characteristics because of the potential time interval between lung cancer diagnosis and survey completion. Further, because this analysis was restricted to respondents with lung cancer from a single year of NHIS, the unweighted sample size for some nonmodifiable demographic subgroups (ethnicity and language) was small. Therefore, we were unable to reliably evaluate associations for these subgroups, limiting our ability to assess potential disparities by ethnicity and primary language. Finally, although survey weights were applied to generate nationally representative estimates, small subgroup sample sizes may limit statistical power for certain analyses.

In conclusion, this nationally representative study demonstrates that current LCS criteria fail to capture a substantial proportion of patients who ultimately develop lung cancer with women being less likely to be LCS-eligible, and that screening uptake remains low even among those who are eligible. Addressing both eligibility gaps and multilevel barriers to screening completion will be critical to improving early lung cancer detection and reducing disparities across populations.

## Supplementary Information

Below is the link to the electronic supplementary material.

Supplementary file1 (DOCX 19 kb)

## Data Availability

Data for this publicly available survey is available at: https://www.cdc.gov/nchs/nhis/documentation/2024-nhis.html.
