# Supplementary material for: Lung cancer screening eligibility and completion among adults diagnosed with lung cancer: retrospective analysis of 2024 National Health Interview Survey
Source: Cancer Causes Control. 2026 Aug 19;37(9):148. doi: 10.1007/s10552-026-02229-x (PMC13490233; doi:10.1007/s10552-026-02229-x)
Supplement: Supplementary file 1 [file 10552_2026_2229_MOESM1_ESM.docx]

**Lung Cancer Screening Eligibility and Completion Among Adults Diagnosed with Lung Cancer: Retrospective Analysis of 2024 National Health Interview Survey**

**Authors:** Izabella Zanazanian BS^1^; Angela Yin Chieh MD, MPH^1^, Aarushi Madan BS^1^, Franceskrista Morales MS^1^, Gelareh Sadigh MD^1^

**Affiliations:**

^1^Department of Radiological Sciences, University of California, Irvine, CA, United States

**Supplementary Table 1.** Univariable and multivariable analysis of non-modifiable patient factors associated with LCS eligibility at the time of diagnosis using survey weights.

**Supplementary Table 2.** Univariate analysis predictors of LDCT prior to lung cancer diagnosis using survey weights.

**Supplementary Table 1. Univariable and multivariable analysis of non-modifiable patient factors associated with LCS eligibility at the time of diagnosis using survey weights.**

| **Characteristics** | **Univariable Analysis**  **OR (95% CI)** | **P value** | **Multivariable Analysis**  **OR (95% CI)** | **P value** |
| --- | --- | --- | --- | --- |
| **Full Cohort**^1^ |  |  |  |  |
| **Sex** |  |  |  |  |
| Female | **0.29 (0.12, 0.72)** | **0.01** | **0.31 (0.12, 0.77)** | **0.01** |
| Male | *Reference* |  | *Reference* |  |
| **Race** |  |  |  |  |
| White | *Reference* |  | *Reference* |  |
| Non-White | **0.31 (0.10, 0.97)** | **0.04** | 0.35 (0.12, 1.04) | 0.06 |
| **Cohort limited to participants who smoke^2^** | | |  |  |
| **Sex** |  |  |  |  |
| Female | 0.62 (0.25, 1.52) | 0.28 | 0.58 (0.23, 1.46) | 0.23 |
| Male | *Reference* |  | *Reference* |  |
| **Race** |  |  |  |  |
| White | *Reference* |  | *Reference* |  |
| Non-White | 0.70 (0.17, 2.82) | 0.61 | 0.67 (1.39, 5.60) | 0.66 |

^1^ Number of observations in multivariable analyses is 111 corresponding to population size of 608,836.

^1^ Number of observations in multivariable analyses is 90 corresponding to population size of 477,858.

Note: Ethnicity and language were excluded from multivariable and univariable analyses because of sparse data (n = 2 unweighted observations for Hispanics in both cohorts; n=5 and 2 for non-English-speaking participants in the full cohort and the cohort with a history of smoking), which would compromise model stability and the reliability of the estimated associations.

Note: Bold represents P value < 0.05.

**Supplementary Table 2. Univariate analysis predictors of LDCT prior to lung cancer diagnosis using survey weights.**

| Characteristics | Univariable Analysis  OR (95% CI) | P value |
| --- | --- | --- |
| **Age** | 0.99 (0.96, 1.03) | 0.59 |
| **Sex** |  |  |
| Female | 0.80 (0.34, 1.88) | 0.60 |
| Male | *Reference* |  |
| **Race** |  |  |
| Non-White | 0.98 (0.36, 2.64) | 0.97 |
| White | *Reference* |  |
| **Language Spoken at Home** |  |  |
| English | 1.10 (0.16, 7.75) | 0.92 |
| Other | *Reference* |  |

Hispanic data is not presented as there were not enough observation to draw conclusions.
